# Clinician and patient perspectives on the ontology of mental disorder: a qualitative study

**DOI:** 10.3389/fpsyt.2023.1081925

**Published:** 2023-05-12

**Authors:** Annemarie Catharina Johanna Kohne, Lukas Peter de Graauw, Reina Leenhouts-van der Maas, Jim Van Os

**Affiliations:** ^1^Department of Psychiatry, UMC Utrecht Brain Centre, University Medical Centre Utrecht, Utrecht University, Utrecht, Netherlands; ^2^GGZ Noord-Holland-Noord, Alkmaar, Netherlands; ^3^Department of Psychiatry, Academic Medical Centre in Amsterdam, Amsterdam, Netherlands; ^4^Arkin (Netherlands), Amsterdam, Netherlands; ^5^GGZ Momentum, Breda, Netherlands; ^6^GGZ De Hoop, Dordrecht, Netherlands

**Keywords:** psychiatric object, diagnostic and statistical manual, thematic analysis, qualitative interviews, typology, recovery, outcome, lived experience

## Abstract

**Background:**

Psychiatry may face an “identity crisis” regarding its very foundations. The lack of consensus regarding the theoretical grounds of psychiatry as a discipline has its epicenter in the discussion about the Diagnostic and Statistical Manual (DSM). A growing number of academics considers the manual broken and a growing number of patients voice concern. Despite the huge body of critique, 90% of Randomized Trials are based on DSM definitions of mental disorder. Therefore, the question regarding the ontology of mental disorder remains: what is a mental disorder, exactly?

**Aims:**

We aim to identify ontologies that live among patients and clinicians, evaluate the degree of consistency and coherence between clinician and patient views and contribute to the establishment of a novel ontological paradigm of mental disorder that is aligned with patients’ and clinicians’ perspectives.

**Method:**

Eighty participants (clinicians/patients/clinicians with lived experience) were interviewed using a semi-structured interview, exploring their ideas on the ontology of mental disorder. This question was approached from different angles which led to comprising the interview schedule into different topics: “concept of disorder,” “representation by DSM,” “what is treated,” “what is recovered,” and “the right outcome measure.” Interviews were transcribed and analyzed using inductive Thematic Analysis.

**Results:**

From all subthemes and main themes, a typology was constructed in which six, not necessarily mutually exclusive, ontological domains were identified: mental disorder as (1) disease, (2) functional impairment, (3) loss of adaptation, (4) existential problem, (5) highly subjective phenomenon, and (6) deviation from social norms. Common ground for the sample groups was that mental disorder is about functional impairment. Although about a fourth of sample clinicians holds an ontological concept of disease, only a small percentage of patients and none of the clinicians with lived experience adhered to an ontological concept of disease. The sample clinicians most often understand mental disorder to be a highly subjective phenomenon, and individuals with lived experience (patients and clinicians) most often understand mental (dis)order to be adaptational in nature: an (im)balance of burden in relation to strengths, skills, and recourses.

**Conclusion:**

The ontological palette is more diverse than what is taught about mental disorder in dominant scientific and educational discourse. There is a need to diversify the current, dominant ontology and make room for other ontologies. Investment is required in the development, elaboration and coming of age of these alternative ontologies, allowing them to reach their full potential and act as drivers of a landscape of promising novel scientific and clinical approaches.

## Introduction

Psychiatry may face an “identity crisis” regarding its very foundations (1). The lack of consensus regarding the theoretical grounds of psychiatry as a discipline has its epicenter in the discussion about the Diagnostic and Statistical Manual (DSM). A growing number of academics considers the manual broken and a growing number of patients voice concern. Many authors observed fundamental problems concerning the limited validity, reliability, loss of information, diagnostic instability and lack of a solid empirical basis [e.g., (2–12)]. Despite the huge body of critique, 90% of Randomized Trials are based on DSM definitions of mental disorder.

Given the critique concerning lack of empirical basis of DSM, it may be considered astonishing that the question about the ontology of the “psychiatric object” is barely discussed or studied. Ontology, in this context, can basically be understood as the existence and being of mental disorder. Or, put in simpler words: our understanding of what mental disorder is, exactly. Our ontological conceptualizations of mental disorder are important because they simultaneously reflect and produce the object they try to represent [see also (13, 14)]. A construction imposed on reality may change reality: it may direct research efforts, determine clinical practice (treatments), determine institutionalizations and physical environments, and may even change the ways in which patients and those around them experience them(selves) (15). The ontology that is held determines what kind of questions are posed in studies, how and what is measured, how we listen to and treat our patients and when we think we should stop treatment. Parnas et al. (16) argue that this neglect of the question regarding the ontology of mental disorder is a crucial source of the stagnation of psychiatric research.

Another reason why the ontology of mental disorder should be studied and debated is because research has repeatedly shown that symptom indicators do not fully capture patients’ and clinicians’ perspectives on recovery [e.g., (17, 18)]. In addition, research has shown that DSM-defined study outcomes based on “symptomatic remission” only partly reflect issues considered important by patients and clinicians (19, 20). If a DSM concept does not fully correspond with the ideas of patients and clinicians at many levels, it may be instructive to examine their ideas on what mental disorder is in more detail. In other words: what ontologies do they hold? And do these ontologies differ between groups? Clinical expertise and values and patients’ wishes are two of the three “pillars” of evidence-based medicine yet the investigation of their ontologies has been mostly neglected to date (21). This can be considered an omission, given the fact that clinicians and patients gather vital information about mental disorder through their daily practices and experiences.

This study aims to further examine this issue by providing a patient and clinician perspective on the ontology of mental disorder. This will help the field to (i) identify ontologies that live among patients and clinicians, (ii) evaluate the degree of consistency and coherence between clinician and patient views, (iii) lay the basis for a new ontological understanding of mental disorder that can serve and steer research and clinical care.

## Methods

### Rational

When explaining a qualitative methodology, many fundamental issues regarding the paradigm by which we pursue science become exposed. Qualitative methodology is often contrasted with and/or a reaction against positivism, the claim of objectivity (realism), reductionism, essentialism, laboratory science, and linear (causal) thinking [see (22)]. To reason and build from its own strengths, however: qualitive research can challenge conventional ideas, inspire new theory, and stimulate minority inclusion and social change (23). As the leading question of this study is comparatively novel, we require an approach that allows an open, deep, and broad exploration. Besides this, we are not sure where we will encounter ideas on the ontology of mental disorder, so a qualitative method may be the best way to approach the research questions. It may help to capture the complexity of the issue better than any other method of research like fixed-choice surveys. In other words, a qualitative method will enable a bottom-up research approach from which new meanings and concepts can be generated. A bottom-up approach, however, does not mean that the stance of the researcher is neutral and without theoretical pre-conceptions. We do not think a point and question from nowhere exists, therefore we explicate it. Our explicit stance derives from relational realism or critical realism, inspired by the thoughts and theories of Latour (24) and Hacking (15). This basically means that we move away from positivism and yet are critical toward pure constructivism. Subject and object are interrelated; scientists (subjects) construct knowledge objects. In the words of Hacking (15), something that is real, is simultaneously constructed. We furthermore hold the idea that psychiatric or psychological science should be built from an explicit ontology. We choose Thematic Analysis as a qualitative research tool as it is a well described and demarcated, yet flexible research tool that has a theoretical freedom and can provide a rich and complex account of data.

### Participants

A total of 80 patients and clinicians from several health care institutions in the Netherlands were enrolled in the study. Recruitment of the 80 patients and clinicians took place at three regional mental health care providers: *De Hoop GGZ*, *GGZ Momentum* and the *University Medical Centre of Amsterdam*. De Hoop GGZ is a mental Health Care institution with a religious (Christian) basis that has locations throughout the Eastern-central part of the Netherlands. GGZ Momentum is a mental Health Care institution with a humanist basis that has locations throughout the South of the Netherlands. This Health Care institution employs a high number of workers with lived experience. The University Medical Centre of Amsterdam is an academic hospital that is based in the Western part of the Netherlands, with an explicit focus on “evidence-based” care.

We recruited clinicians via the professional networks of AK, LDG, and RL. As participating in this study would entail a time investment for clinicians who already have very full calendars, we chose for this form of convenience sampling. With the use of snowball sampling, we included patients (these were the (ex-)patients of the clinicians we interviewed) and more clinicians and their (ex-)patients. All clinicians were selected based on “purposeful selection”: we included them based on the expectation that they were able to provide in-depth and detailed information about the ontology of mental disorder (25). In addition, we used two criteria for including clinicians in this study: they (i) were seeing patients for diagnosis, treatment, or both and they (ii) were licensed to practice under the Dutch BIG-register. In this way a proper level of training and education could be assumed.

Inclusion of patients was based on their motivation to participate in the study. There was no explicit exclusion criterion, although patients that were in the first stages of their treatment were not actively approached. No participant compensation was provided. Travel expenses were reimbursed if needed, but none of the participants asked for reimbursement (possibly because of the online option that emerged because of the COVID-19 pandemic). The final sample consisted of three groups: patients, clinicians (registered mental health care professionals) and individuals who had been both clinician and user of mental health services.

### Procedure

Over the course of 2 years, interviews were conducted with participants, using a semi-structured interview schedule. We chose a semi-structured format to leave room for new, other, and emerging ideas of participants during the interview. Interviews were audio taped, and typically lasted between 60 and 90 min. Interviews took place in Dutch.

The leading question of this investigation was approached from different angles which led to various interview topics. The semi-structured interview schedule was divided into five main topics: (i) concept of disorder (what exactly is mental disorder?); (ii) treatment (what is treated and how is it (best) treated?); (iii) recovery (what is recovery and what is recovered?); (iv) DSM (does a DSM-diagnosis represent a mental disorder?) and (v) outcome measure (what is the right outcome measure in our field?). A translation of the Interview Schedule can be found in Supplementary Table S1.

Participants were informed and recruited about the research by e-mail. In the case of patient inclusion this was done either after their treatment or at the final stage of their treatment. Those who were interested were given an information letter and provided an opportunity to ask questions about study and procedure. Prior to the start of the interview, participants signed an informed consent form. Audio files were made and carefully locked away for an agreed period of time (typically 2 to 4 weeks). Within this agreed period of time, an anonymized transcript was generated after which the audio file was deleted. Information that could reveal the participant’s identity was removed from the transcripts. All transcriptions of audio files were exported to a protected hard drive at Utrecht University Medical Centre. The study was run after review of and approval by the Medical Ethics Committee and the Institutional Review Boards of the participating health care institutions. Interviews took place at the location of the participant’s choosing (either at the health care provider or, because of the outbreak of the COVID-19 pandemic, online).

### Analysis

The analysis consisted of three main steps. In the first step, the focus was on familiarization with the data. Transcripts were read several times and a mind-map of each interview was constructed by one of the researchers who did not conduct the interview. This was done to grab each participant’s core concept of ontology by reading and seeing the whole of the participant’s story. After this, the researcher who conducted the interview checked the mind-map for accuracy. Consensus over findings and interpretations of the mind-maps was reached by discussion.

The second step involved a more thorough analysis of the transcripts using inductive thematic analysis (26). The Qualitative software analysis program MAXQDA was used to assist with the analyses. Initial codes were generated after which transcripts were analyzed a second time, noting emerging and overlapping themes (repeated patterns). We primarily adopted a semantic approach in which the themes were identified within the explicit meanings of the data (26). Transcripts and emerging themes were reviewed by two additional researchers in order to provide external validation of the analysis. Both systematically checked all codes. This ensured that the themes were represented in the source material. In case of discrepancies, consensus was reached by discussion. Main themes and subthemes were identified, reviewed, refined, and ultimately defined. Although more instances of a theme across one interview and across the whole dataset does not necessarily mean the theme is more substantial, the focus was on themes with a certain weight and frequency across the entire data set (frequency > 10 times). This was done to make sure to not capture idiosyncratic conceptualizations but capturing ideas that live more broadly in the field. These steps resulted in the identification of various themes and an overview of the frequencies of these themes across the whole of the dataset as well as per participant group. In this step, we also decided to omit the topic of “representation by DSM.” Although that the topic is considered highly important, we discovered that the topic was too broad and encompassing too many areas, defying meaningful synthesis for the purpose of answering the leading question of the study.

In the third step, we looked for patterns in the themes across all topics that we identified in the previous step. We looked for the overarching ontological conceptualizations that would answer our leading question. This led to the “identification-construction” of six overarching ontological domains that will be presented in a typology in the results section. We finally checked these over-arching ontological domains with all mind-maps, in order to examine if the typology captured and included the core concept of ontology of participants. Finally, the embedded findings and interpretations were discussed by all authors.

## Results

### Participant characteristics

Between September 2018, and December 2020 a total of 80 participants were enrolled in the study who all consented to participate [registered clinicians *N* = 37 (46%); patients *N* = 22 (28%); clinicians with lived experience *N* = 21 (26%)]. Among the registered clinicians 19 (51%) were women and 18 (49%) were men. The mean age of the clinician group was 43.9 (SD 11.4) years. Among the group of registered clinicians 12 (32%) were psychiatrists and 27 (68%) were psychologists. The ratio of psychiatrists to psychologists is about 1:5 in the Netherlands. Of the patient group, 12 (55%) were women and 10 (45%) were men. The mean age of the patient group was 39.7 (SD 11.7) years. Among the clinicians with lived experience of mental health treatment 16 (76%) were women and 5 (24%) were men. The mean age of the clinicians with lived experience of mental health treatment was 34.4 (SD 11.6) years. All three groups had a higher proportion of women than men. During the interview it transpired that several clinicians received treatment for mental distress in the form of psychotherapy/pharmacotherapy. These individuals were placed in the “clinician with lived experience” group. We did not ask about the nature of their condition but let them choose to share this information or not. Clinicians were not placed in the lived experience group if they had sought other forms of help such as coaching, meditation retreats, etc. Table 1 displays the participants’ characteristics.

**Table 1:** Participant characteristics.

Clinicians		Clinicians with experimental knowledge		Patients	
Number (*n* = 37)		Number (*n* = 21)		Number (*n* = 22)	
**Gender**		**Gender**		**Gender**	
Woman	19 (51%)	Woman	16 (76%)	Woman	12 (55%)
Men	18 (49%)	Men	5 (24%)	Men	10 (45%)
**Age**		**Age**		**Age**	
Mean (SD)	43.9 (11.4)	Mean (SD)	34.3 (11.6)	Mean (SD)	39.7 (11.7)
Range	30–71	Range	25–54	Range	21–65
**Profession**		**Profession**		**Employment status**	
Registered Psychiatrist	12 (32%)	Registered psychiatrist	1 (5%)	Employed	11 (50%)
Registered Psychologist	27 (68%)	Registered psychologist	15 (71%)	Not employed	3 (14%)
		Other (psychologist/nurse/social worker)	5 (24%)	Other (retired, student or volunteer)	8 (36%)
				Unknown	3 (14%)
**Current workplace** *		**Current workplace** *			
Hospital	16 (43%)	Hospital	1 (5%)	**Received treatment (current)**	
MHC Institution (GGZ)	21 (57%)	MHC Institution (GGZ)	20 (95%)	MHC Institution (GGZ)	22 (100%)
Private practice	2 (5%)	Private practice	1 (5%)		
**Past workplace(s)** *		**Past workplace(s)** *		**Received treatment (past)**	
Hospital	13 (35%)	Hospital	2 (10%)	Hospital	6 (27%)
MHC Institution (GGZ)	28 (76%)	MHC Institution (GGZ)	12 (57%)	MHC Institution (GGZ)	13 (59%)
Private practice	2 (5%)	Private practice	4 (19%)	Private practice	11 (52%)
Other	10 (27%)	Other	4 (19%)	Unknown	6 (27%)
Unknown	1 (2%)	Unknown	2 (10%)		
		**Condition** *		**Condition** *	
		Anxiety disorder	4 (19%)	Anxiety disorder	12 (54%)
		Mood disorder	5 (24%)	Mood disorder	6 (27%)
		Addiction or behavioral problems	3 (14%)	Addiction or behavioral problems	10 (45%)
		Eating disorder	9 (43%)	Eating disorder	3 (14%)
		Personality disorder	3 (14%)	Personality disorder	5 (23%)
		Other/unknown	5 (24%)	Other/unknown	3 (14%)

* Multiple answers possible.

### Inductive thematic analysis

The inductive thematic analysis of the 80 interviews resulted in 52 subthemes (bottom-up data patterns), 23 main themes (patterns in subthemes) and 6 overarching domains (pattern over all main themes). The domains will be presented in the next section, the main themes per topic are shown in Table 2 below and all 52 subthemes can be found in Supplementary Table S2. Ontologies were most explicitly expressed by participants when asked about conceptual issues (“what is mental disorder?”), however their ontology was also expressed when discussing other topics. Ontologies were often stabile across the interview: interviewees typically held a concept of mental disorder that was also present in their ideas on treatment, recovery, and outcome. If, for example, a participant held that mental disorder is a highly subjective phenomenon, he/she would often also express that he/she would treat a subjective and personalized goal, that recovery is a subjective process and that the right outcome measure should be subjective and personalized in nature. Participants typically held two or sometimes three ontologies that co-existed harmoniously together (e.g., mental disorder as a loss of adaptation and as functional impairment).

**Table 2:** Inductive thematic analysis of topics with identified main themes, theme frequency.

Topic	Main theme	Freq theme	Freq participants (%)
Concept	Illness	14	11 (14%)
	Impairment functioning	157	61 (76%)
	Adaptational problem	50	30 (38%)
	Deviation (social) norms	63	24 (30%)
	Existential problem	49	24 (30%)
	Subjective phenomenon	49	40 (50%)
Treated	Disease	16	13 (16%)
	Adaptation and functioning	40	30 (38%)
	Existential problem	54	34 (43%)
	Subjective-personalized	48	34 (43%)
	Self-other relations	27	22 (28%)
	Source/core	33	28 (35%)
Recovered	Clinical recovery	115	51 (64%)
	Recovery of functioning	109	54 (68%)
	Recovery as improved adaptation	79	49 (61%)
	Existential recovery	173	55 (69%)
	Recovery as subjective phenomenon	81	39 (49%)
	Personal recovery	82	40 (50%)
Outcome	Syndromes, symptoms	20	19 (24%)
	Improved functioning	45	37 (46%)
	Improved adaptation	28	23 (29%)
	Existential change	73	41 (51%)
	Subjective, personalized outcome	95	49 (61%)

This was, however, not always the case. Sometimes seemingly inconsistent ontologies were identified within one interview. For example, one interviewee held an ontological concept of a “diseased brain,” but defined recovery as “a process of acceptance, growth, and gaining autonomy.” At the group level, other interesting inconsistencies were present. As shown in the participant frequencies per topic (Table 2), a substantial proportion of participants held a concept of “deviation from social norms,” yet this fundamental idea on what mental disorder is did not translate into ideas about treatment, recovery, and outcome. Another interesting observation from participant frequencies per topic is that recovery was defined quite broadly and inclusively compared to the answers on other topics. Thus, participants named many things when asked about what recovery is (e.g., reduction of symptoms as well as being able to function as well as finding one’s narrative as well as a highly personal trajectory). Thus, at individual as well as group level, ideas at the level of concept most often resonated with ideas about treatment, recovery and outcome or the other way round, but this was not always the case. With these data, we cannot tell whether practice translated into concepts or the other way round, but we observed consistency in most instances.

## A typology of the ontology of mental disorder according to patients and clinicians

**Figure 1:**
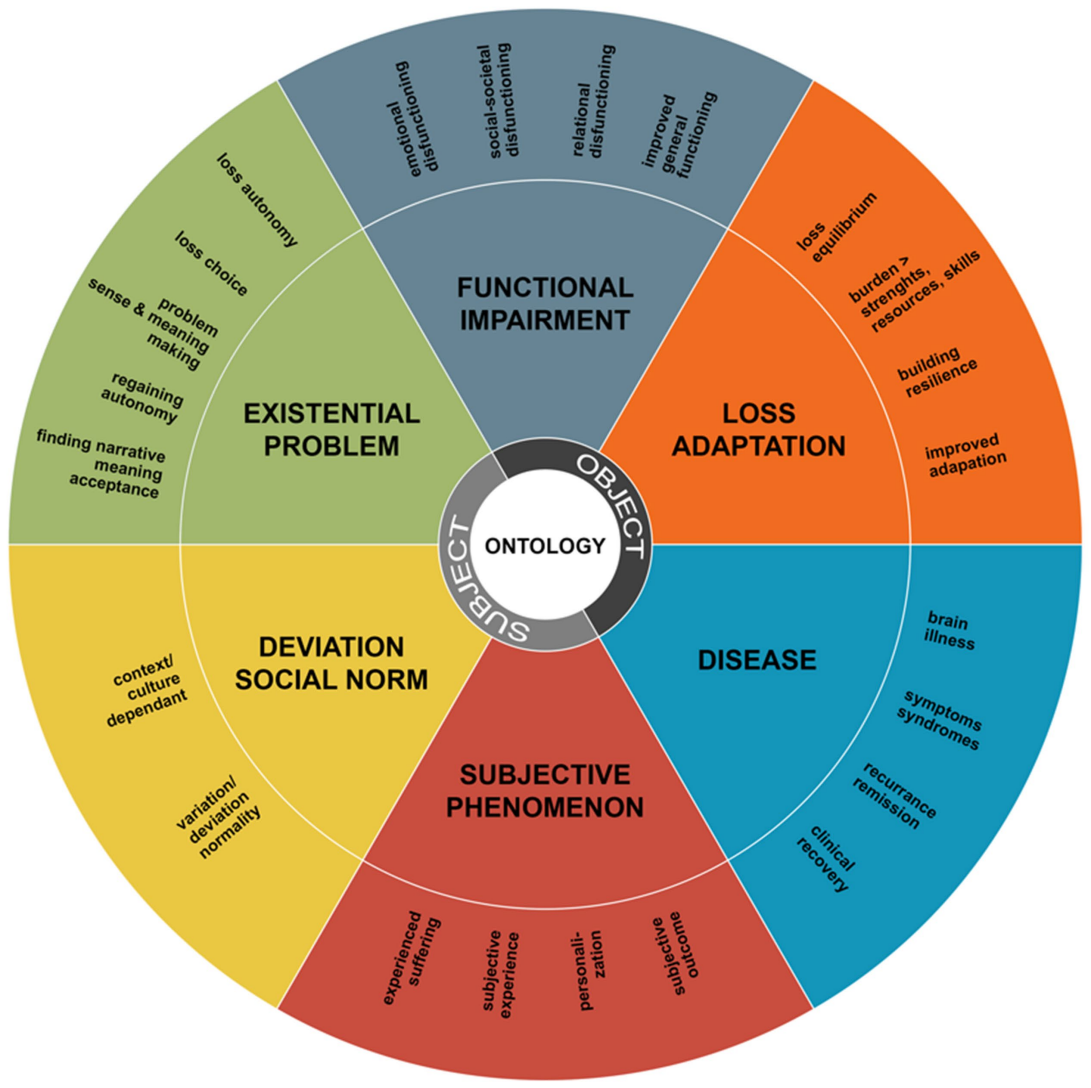
A typology of the ontology of mental disorder according to patients and clinicians, not to be read as a static or quantitative classification. The themes that showed over all topics and that finally formed the six ontological domains are displayed in the outer circle (concept of disorder, that what is treated and recovered and the right outcome measure). The ontological status in terms of reality representation can be found in the inner circle (subject-object). The six domains are displayed in the central ring.

The inductive thematic analysis of the interviews led to the identification of overarching themes—called ontological domains—that represent the ontologies of mental disorder that lived among clinicians and patients. We looked for relations, overlap and patterns in all subthemes and main themes and could identify six ontological domains: (1) mental disorder as disease, (2) mental disorder as functional impairment, (3) mental disorder as loss of adaptation, (4) mental disorder as existential problem, (5) mental disorder as highly subjective phenomenon, and (6) mental disorder as deviation from social norms. We constructed a visual typology from these final six domains (Figure 1) that should not be read as a static classification of reality, but rather as a typification of the different ways in which clinicians and patients understand the ontology (being and existence) of mental disorder. Below, we will describe each of the six ontologies that “emerged/was constructed” from the data. The ontologies were most explicitly and clearly expressed by participants when asked about concept of disorder (“what is mental disorder?”) so the frequencies that are discussed in this section are based on frequencies of this topic.

As shown in Figure 1, the first, inner ring is a typification of the reality representation of the ontologies. Half of the ontologies were more realist and objectivist in their account, while the other ontologies were more constructivist and subjectivist in their ontological account. Below this will be described in more detail for each ontological domain. In the last, outer ring the themes that shaped the domains are displayed. This outer ring contains themes regarding concept of disorder, that what is treated, that what is recovered, and concept of outcome measure. The ontological domain of “deviation from social norms” forms an exception to this, as this ontology only showed when participants were asked about their concept of disorder. Despite this fact, we consider it to be one of the key domains as mental disorder was clearly and explicitly understood as a “deviation from social norms.”

In Table 3, a set of illustrating quotes of the ontological domains are displayed. We translated all excerpts from Dutch to English. The quotes that were selected represented the ontological domains best, although many more representative quotes are available. The quotes are presented to give a clearer and more lively depiction of the ontological domains. The quotes give a narrative presentation of the findings and show elements of the living worlds of participants. We think this is very important as we wish to bring context, details, and nuance to the story.

**Table 3:** Quote table with illustrating quotes of the six ontological domains.

Disease	“Yeah, so those are serious mental illnesses, where um… where the basis is in the brain, so it is, it is basically a brain disease (C, Female, 44, Psychiatrist); Of those very serious syndromes, I am sure that someday they will find something in the brain, or that someday they will understand how that works as a disease of the brain. (C, Male, 32, Psychiatrist); I personally find someone best recovered when I think the complaints that someone came in here with and for which they sought help from me have largely faded. Or are subclinical. (C, Male, 43, Psychiatrist); Sufficient symptom reduction […] and do not make the mistake of, ‘yes but, uhm… The patient is not better yet.’ No, the patient is not better, no. (C, Male, 66, Psychiatrist)”
Functional impairment	“I think functioning, really purely the practical functioning. Yeah. In society… [that’s a] very good measure of how you feel inside yourself. (P, Male, 28, Gilles de la Tourette, ADHD); How you describe a disorder I find very difficult… Yeah, that is the feeling that I do not function as I would like to. (EC, Female, 52, Social Worker, Anxiety disorder); But recovery for me equals normal functioning… I think for myself a treatment would almost be kind of a failure if I do not function normal in the long run. So just go to work… (P, Male, 44, Alcohol Addiction, Anxiety Disorder, Personality Disorder). I think ultimately you have to start looking at um… How someone’s functioning improves […] in the work area, in the relational area. (C, Male, 37, Psychiatrist)”
Adaptational problem	“We always strive for balance, but you have certain equilibria that are more sickening, so to speak […] And certain balances that are a little more… shall I say, helpful. Or involve a little less suffering. (C, Male, 37, Health Care Psychologist); It is not, of course, that complaints are always reduced to zero […] The recovery process is that you can accept that and that you know how to compensate for that with all kinds of healthy facets in life (P, Male, 43, PTSD); Suppose you have 2 sisters who grow up in the same environment and they both have more or less the same anxious disposition. Eventually they break up and one of them gets into a very supportive relationship, then the environment, such a supportive thing, does determine very much whether it develops into a disorder. (C, Female, 33, Health Care Psychologist); And yes, I still feel like shit quite often but that’s okay, I feel skillful. And that’s what the treatments have done for me. So, I think that’s actually and may be the essence. (P, Male, 42, Personality Disorder, Addiction, ADHD, PTSD)”
Deviation social norms	“A mental disorder, as far as I am concerned, is an um… Significant deviation from the normal distribution in society, concerning thinking, doing, and/or feeling, for which there is subsequently no place in society. (C, Male, 57, Clinical Psychologist); For me, it has always been that I felt very disconnected from society and the norms of others. Because I was behaving differently and not understanding why at that point. Before I knew that I have a mental disorder. (EC, Male, 26, Social Worker, Bipolar disorder, Addiction); I see it mainly as a kind of joint definition of what we consider deviant in a certain time at a certain place. (EC, Female, Health Care Psychologist, Somatoform disorder NOS)”
Existential problem	“Clients recover when they feel autonomy, have their own choice in life. I decide, I sit at the wheel, I choose. Instead of ‘because the world has hurt me so much, I do this’. (EC, Male, 28, Social worker, Addiction); That you feel like you are at the wheel of your own life actually. Also experiencing self-management about the complaints and limitations that you may still have and dealing with them. And experience both autonomy and connection, because I have the idea that these are really two key concepts in recovery. (EC, Female, 38, Psychiatrist, condition unknown); A narrative is not only very important for understanding and accepting what happened to you…but a story is often also about strengths and beautiful qualities. By understanding the story, you can sometimes look at yourself with more mildness […] and start to reflect on alternatives. (C, Male, 41, Psychiatrist)”
Subjective phenomenon	“It is very subjective, I think, where you can have a physical complaint very clearly examined by a doctor whether it is there or not or how big your tumor is or whatever, a psychological disorder is very subjective. And very complex because it is related to so many things. (EC, 30, Female, Psychologist, eating disorder); I do not think you can measure it…when I think of research…it has to be quantifiable. I find that very difficult to impose on someone. One person’s recovery is not a recovery for the next person. (P, Male, 49, Social Anxiety Disorder and Alcohol Addiction); One patient is already satisfied if he eh, if he has sleeping problems that he sleeps an extra 15 min. And the other patient is only satisfied if he has… five extra hours of sleep. Yes, and that depends on everyone personally. (C, Male, 66, Psychiatrist); I cannot tell what recovery is and what not because I think it is really so incredibly personally bound what recovery is. No, so I do not think there is one fixed answer [regarding outcome]. (EC, Female, 28, Health Care Psychologist in training, Eating Disorder)”

C, Clinicians; EC, Clinicians with lived experience; P, Patients.

### Mental disorder as disease

The first kind of ontological conceptualization that was found, is well known and can be best described as a “medical-disease conceptualization.” Mental disorder is understood as a thing that typically resides and/or originates in the brain of the individual. In that sense it is an objectivist, realist account in which mental disorders are understood as natural kinds that are real entities that can be objectively observed. Language that typically accompanies this kind of ontological conceptualization includes words like “illness,” “brain-disease,” “biological substrate,” “symptoms,” “response,” “remission,” “symptom reduction,” “symptomatic remission,” and “clinical threshold.” It is often described as something that is determined and static in spatial and temporal terms. Interestingly, in this ontological stance some interviewees differentiate between psychiatric disorders and psychological disorders in which psychiatric disorders are understood as illnesses of the brain and psychological disorders are understood as problems of living [see correspondence to the findings of Ahn et al. (21)]. Within participants, this ontological conceptualization of mental disorder often co-existed with an ontological conceptualization of functional impairment. Here, the criterion for “mental disease” lies in the impairment of functionality or the underlying mental disease can be measured by functional-behavioral impediment. The focus for treatment and recovery in this ontology is remission of the illness/symptoms. Clinicians (psychiatrists) from the university medical center formed the heart and majority of this group. Although there were not many participants that understood the ontology of mental disorder to be a disease, there were many participants that understood recovery as “learning to live with” or self-management of symptoms.

### Mental disorder as an impairment in functioning

The second kind of ontological conceptualization of mental disorder that was found, can be described as a “functional” ontology of mental disorder. The principle of usefulness or functionality is applied to signify the nature of mental disorder. This ontological stance can be understood as realist and pragmatist as mental disorder is viewed as a problem of functional-practical nature that is objectifiable, real and can be measured. Some participants stressed social-societal or professional roles as most important. Others stressed relational functioning as key, whereas other interviewees underlined emotional or mental functioning as most important. Most often, participants spoke of “general functioning” in which they combined several of these aspects. The beginning and endpoint of this ontological conceptualization was often understood in behavioral terms. The concept and outcome were often defined as (not) being able to “fulfill one’s roles,” “have a work life and social life,” “do what you need or want to do” etc. The larger part of the participants from each of the groups held some sort of “functional” ontology.

### Mental disorder as adaptational problem

The third kind of ontological conceptualization of mental disorder that was found, can be described as an ecological or adaptational ontology. Adaptation refers to the adjustment of humans to become more suited to their environment. In a situation of imbalance by burden, adversity or vulnerability, humans may successfully adapt by finding resources of support and strengths, and by building skills to regain equilibrium and resilience. Examples that were given are improving regulation skills (i.e., self-regulation, emotion-regulation) or finding a suitable and supportive partner, work and friends. People are thought to be able to work on either front; they can improve strengths, recourses and skills or they can lessen their burdens. This ontological conceptualization explicitly incorporates context and fit to context and is systemic in that sense. In this type of ontology, mental disorder is thus conceptualized as not finding or regaining a healthy equilibrium: an imbalance between resources and vulnerabilities remains. This ontological stance can be understood as realist and naturalist as mental (dis)order is conceptualized as a dynamical (eco)system that follows the logic of nature. Although complex, mental disorder is thought to be objectifiable, real and measurable. Imbalance of equilibrium is the point of beginning (concept) and regaining equilibrium is the endpoint (outcome) of this ontological conceptualization. The ecological or adaptational ontology was held by almost half of the sample participants with lived experience and by around a third of the sample clinicians.

### Mental disorder as deviation from social norms

The fifth kind of ontological conceptualization of mental disorder that was found, can be described as a “contextual deviation” ontology. This ontological stance can be contrasted with the disease conceptualization in the sense that it (i) places the problem not within an individual but in (between) the context (and an individual), (ii) understands mental disorder not as an illness of the brain but as a deviation from and product of sociocultural norms. This ontological understanding holds that within the small, non-inclusive norm within society, there is no place for variation or deviation. Mental disorder is a product of societal norms; the non-inclusive society creates further deviation mediated by exclusion and loneliness. This ontological stance can be understood as social constructivist and relativist as mental disorder is conceptualized as a “variation” that is produced by society itself. Socio-cultural norms are the point of beginning (concept), yet only very few interviewees (*n* = 2) mentioned a change of our sociocultural norms as the right endpoint (outcome). Many times, though, the solution to the problem was thought to be within the individual although the problem itself was thought to lie in society. Although around a third of each of the groups held a core concept of social deviation, it was hardly ever the answer they provided when asked what was treated, recovered or what they viewed as the right outcome measure.

### Mental disorder as existential problem

The fourth kind of ontological conceptualization of mental disorder that was found can be described as an “existential” ontology. This ontological stance incorporates the concepts of autonomy, narrative, self-governance, self-reliance, sense- and meaning making and freedom of choice. This ontological stance encompasses the idea that mental disorder is about not being able to govern your own life and live according to your own values. In this ontological conceptualization, mental disorder is an experience of loss of autonomy, choice, and meaning over existence. This stance encompasses the idea that the narrative you hold about yourself and live are toxic in the case of mental disorder. In that sense it is a subjectivist, constructivist account in which mental disorders are understood as human constructs that reside in the mind of individuals. In this ontological conceptualization, the point of beginning may be best defined as existential disconnectedness (concept) and the endpoint (outcome) is growth by acceptance and change of meaning and narrative. About a third of patients and clinicians held this ontological stance and about a fifth of clinicians with lived experience held mental disorder to be an existential problem. Although it may be expected that participants from the Mental Health Care institution with a religious (Christian) basis largely formed this ontological stance, this was not the case. Participants that understood mental disorder to be existential in nature were evenly distributed over the three included mental health care institutions.

### Mental disorder as subjective phenomenon

The sixth kind of ontological conceptualization of mental disorder that was found, can be described as a “subjective-phenomenological” ontology. This stance is non-essentialist at hearth for it holds that mental disorder is a highly subjective phenomenon that differs from disorder to disorder and from person to person in its experience, shape, color, etc. (qualia). In that sense it is a subjectivist, constructivist account in which mental disorders are understood as highly personal phenomena that reside in the minds of individuals. In this ontological stance quantification and objectification of the phenomenon is questioned, if not thought impossible. The subjective feeling of suffering is the point of beginning of this ontological conceptualization and the endpoint (outcome) is highly subjective and personalized in this stance. About a third of patients and clinicians with lived experience held this ontological stance and about two thirds of clinicians held this ontological stance.

### Differences between patient and clinician perspectives

**Figure 2:**
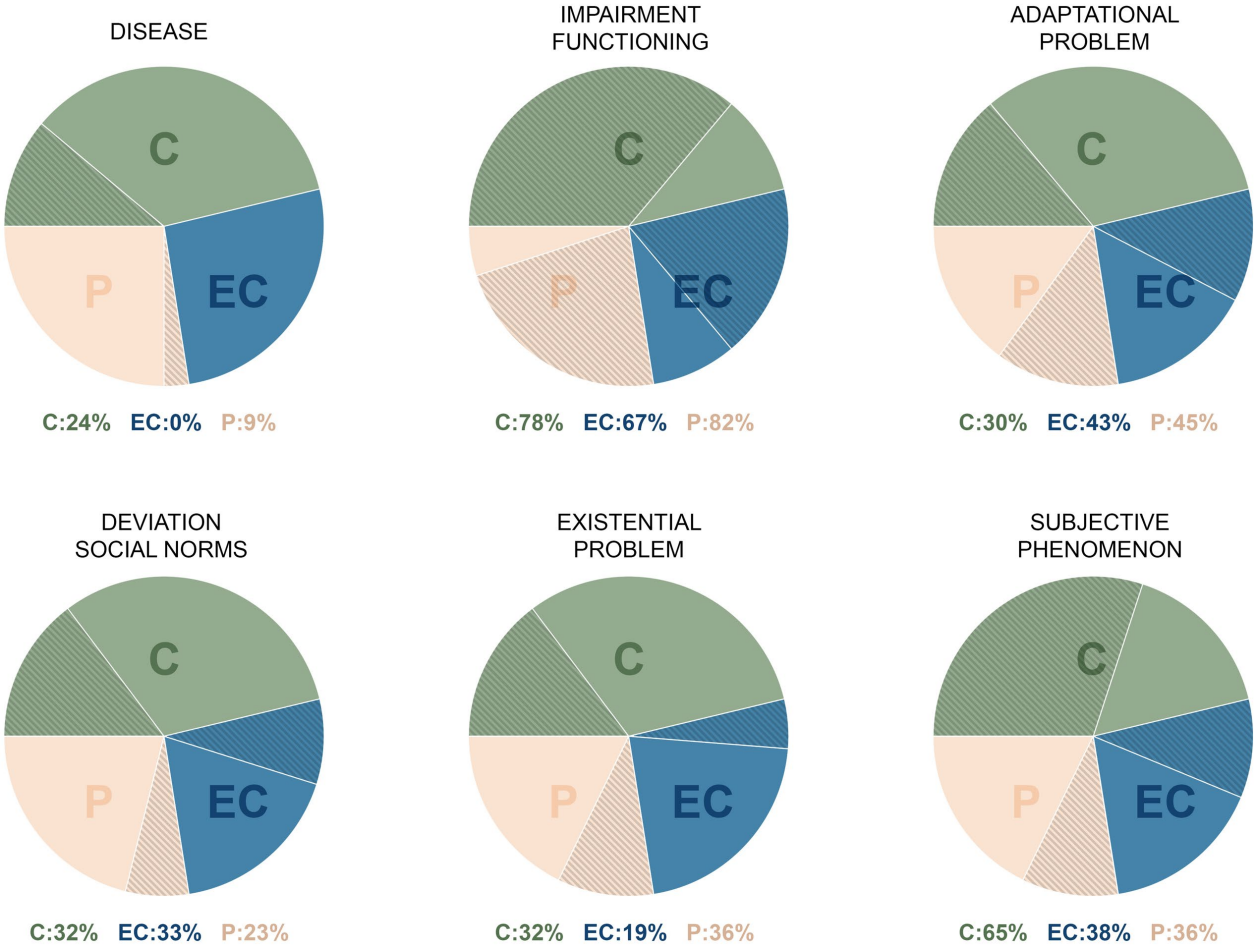
Impression of subgroup percentages of participants (against background of total subgroup) per ontological concept. This visualization is based on the frequencies of “concept of disorder.” C, Clinicians; EC, Clinicians with lived experience; P, Patients.

In order to give an impression of the differences between sample subgroups in their ontologies, we created a visualization (Figure 2) of subgroup frequencies per ontological domain. Common ground for the three groups in the sample, in terms of ontology, is that mental disorder is about functional impairment. This means that many of the participants from our sample understand mental disorder—at least in part—as functional in nature. However, at the group level, there are some interesting differences. As seen in Figure 2, about a fourth of sample clinicians holds an ontological concept of disease. However, only a small percentage of sample patients and none of the sample clinicians with lived experience adhered to an ontological concept of disease. Also shown in Figure 2, a majority of sample patients and clinicians with lived experience holds an ecological-adaptational ontology, yet a majority of sample clinicians holds a “subjective-phenomenological” ontology. Thus, the clinicians most often understand mental disorder to be a highly subjective phenomenon and individuals with lived experience (patients or clinicians), most often understand mental (dis)order to be about (im)balance of burden and strengths, skills, and recourses. Both groups think that mental disorder is, to some extent, about functional impairment. Within the sample subgroups, many differences in ontological stance are present. This gives rise to the question what this and the above implies for clinical care, research, policy and maybe even education.

## Discussion

This contribution aims to emphasize the importance of questioning and explicating the “psychiatric object” and introduces one of the first studies into patient and clinician perspectives on the ontology of mental disorder. In sum, the analyses show a broad ontological palette provided by patients and clinicians and show that these groups differ in their ontological conceptualizations.

A very fundamental observation from our study is that the ontological palette is more diverse than what is taught about mental disorder in dominant scientific and educational discourse (In this case: DSM, linear medical model, bio-psycho-social-model). The implicit ontology that we are taught and instructed with does not fully cover the ontological conceptualizations of people with lived experience and clinical-practical knowledge. This fundamental finding implies that the current, dominant ontology may require modification to make room for other ontologies. If we wish to do justice to all the three “pillars” of evidence-based medicine, ideas on ontology that are held by clinicians and patients need to be taken into account. This means that we need to invest in the development, elaboration and coming of age of these other ontologies. If these ontologies will reach their full potential, they may create a landscape of promising novel scientific and clinical approaches in mental health care and research.

Examining the findings, we see many differences in the ontological concepts of the participants. We may hypothesize that ontological match-making may be crucial for treatment success, parallel to the suggestion that treatment of mental problems will be more successful if patient and professional concur on the underlying explanatory model and theory of change (27). If, for example, Amy wants to understand and rewrite her own life story to heal her suffering, she will feel more lonely and less heard when her psychiatrist and psychologist prescribe antidepressants and behavioral therapy. Likewise, the other way around. If Bob thinks an illness has befallen him, and he wants antidepressants, he will become frustrated if his psychologist or psychiatrist wants to talk about the early relationship with his parents. Even if clinicians have another idea about what is going on with their patient than the patient herself, they cannot deny that differences in ontological concepts between themselves and their patients to be a stand-alone negative effect (e.g., lack of adherence, decline trust and compliance). The problem is that the ontologies that are held by clinicians and patients are often implicit and under the table, although we may gain a world if we at least explicate and discuss them. A question for further research is what it means for the structure and dynamics of our systems and institutions that we differ in our perceptions of the psychiatric object, as the differing ontologies may imply that certain kinds of mental health care are not available at some places or institutions.

Besides the fact that ontologies seem to differ between participants, our sample groups also showed some interesting differences between them. A first observation is that a quarter of sample clinicians held an ontological concept of disease whereas only 10% of sample patients and none of the sample clinicians with lived experience held this ontological concept of disease. From a pure realist paradigm, one could argue that this is the case because we did not include individuals with “real psychiatric disorders.” We can, however, glance from the sample characteristics that this is not the case. Many individuals with lived experience received specialist care reserved for severe mental illness, many had comorbidity and about 10%–30% of individuals with lived experience received treatment at the hospital, which all point in the direction of a degree of severity. From a pure realist, disease paradigm, a clinician will argue that illness awareness may be lacking. If we, however, examine this finding from a subjectivist paradigm, we must conclude that people with lived experience barely recognize themselves in illness conceptualizations. In most instances, they do not see their mental disorder as an illness. Often, however, at the group level, they hold an ecological-adaptational ontology which is about the (im)balance of burden and strengths, skills, and recourses. The question that rises is whether we should stick to an illness concept in our dominant ontology and corresponding discourse if this is not attuned to the perspectives of people with lived experience and only to a quarter of the perspectives of clinicians. We think these results may indicate that we should adjust our ontological concept of mental disorder toward a dominantly adaptational-ecological ontology. We may envision a form of health care in which patients can work on either side of the balance: strengthening skills and recourses or working on burdens/problems/pains.

Furthermore, a large number of sample clinicians and individuals with lived experience think that mental disorder is highly subjective in nature; they ultimately think we need personalized *N = 1* theories. If we take this ontological stance one step further, we can imagine replacing science based on group-level outcomes that are applied at the individual level, by a science based on individual cases (personalized psychiatry) that may lead to interventions at the group level (28). Another ontology that showed itself in this study, is existential in nature. We are hopeful that the existential domain will get more attention in the field of psychiatry. Indeed, existential fear is formulated as a transdiagnostic factor, for which treatment programs may be developed [e.g., (29)].

Finally, an interesting finding was that mental disorder is quite often (about 30% across all groups) understood as a deviation from social norms. Although it was their ontological concept, only very few interviewees mentioned a change of our sociocultural norms as the right endpoint; it was hardly ever the answer they provided when asked what was treated, recovered or what they viewed as the right outcome measure. The solution to the problem was thought to be within the individual although the problem itself was thought to lie in society. We think this is an important finding because if we take this ontology of mental disorder seriously, we should think about solutions that transcend the individual and are directed at society and the norms that live within society. In fact, this ontology may plea for prevention efforts and public health interventions that target stress, exclusion, social injustice, etc. (30). It may also imply that treatments should be targeted at systems (e.g., families, schools, etc.) instead of individuals. It may even mean we will need a cultural change in our societies. This ontological understanding of mental disorder necessarily places responsibility on society and politics. As the demand for mental health care is rising, further studies on the demarcation of our object and work seems vital.

### Limitations

Besides these fundamental reflections, several methodological limitations of the current study should be taken into consideration. First, from the pattern in the data, we moved to hypothesis and theory. Therefore, our conclusion drawn from this inductive method cannot be “proven” and remains an educated guess. However, for a qualitative study, we did include quite a large number of participants which makes our findings and conclusions more representative.

The second limitation of this study may be the operationalization. We included clinicians from the hospital and only two secondary mental health care institutions from the Netherlands. In addition to this, we only included patients from secondary mental health care institutions as patients from the hospital were often in the stage of crisis or at their first stages of treatment. One may consider this as an operational limitation as the included participants may not be representative of the ideas that live in their group and in the field; they may be considered to speak from a specific context, organizational culture, or limited experience.

However, as can be seen from the sample characteristics, all clinicians and patients were asked about their past workplaces and past treatments. In these past workplaces and past treatments, most often a very diverse experience is present. Further, the Netherlands may be considered a liberal society and all interviews were anonymous, so we think that participants felt the liberty to think for themselves and from this wide experience. Therefore, this operational aspect likely will not represent a major problem.

We nevertheless think it is important to replicate the current findings in other and more samples, as to test the presented ontological typology. In future research, it may be interesting to look at gender differences and study other and more samples to be able to compare the ontological ideas of different diagnostic samples (e.g., persons with psychotic disorders vs. anxiety disorders). In addition, it would also be interesting to look at moderators like, for example, diagnosis, gender, or ethnical background. Although the findings may not be conclusive, this study does show that unexplored and underserved perspectives on the ontology of mental disorders live among patients and clinicians. Policymakers should invest in the development, elaboration and coming of age of these other ontologies. Educational discourse should at least openly discuss the controversy around the psychiatric object and show and discuss this broader ontological palette. Educational discourse should be more inclusive regarding differing opinions. We need collaborative innovation in the field of psychiatry in which clinicians and patients co-create ideas on the “psychiatric object” that may drive and steer a landscape of promising new approaches. For clinical practice, this may ultimately and hopefully translate into more transparent dialogs and attuned treatment efforts.

## Conclusion

This contribution introduces one of the first studies into patient and clinician perspectives on the ontology of mental disorder. The current, dominant ontology that is taught and instructed does not fully cover the ontological conceptualizations of people with lived experience and clinical-practical knowledge; they have other and novel ways of understanding the “psychiatric object.” The DSM has committed us to a one-dimensional and poor ontology that has major implications for clinical practice and all stakeholders involved. The fundamental findings of this study imply that we need to consider modifying the current, dominant ontology and make room for other, complementary ontologies. If we wish to do justice to the three ‘pillars’ of evidence-based medicine, we must invest in the development, elaboration and coming of age of the ontologies on mental disorder that are held by clinicians and patients. For the field to progress, we need collaborative innovation in the field of psychiatry in which clinicians and patients co-create ideas on the ‘psychiatric object’ in order to develop new avenues of research and practice.

## Data availability statement

The raw data supporting the conclusions of this article will be made available by the authors, without undue reservation.

## Ethics statement

The study protocol was approved by the Medical Ethics Committee of the Academic Medical Center of Amsterdam and the Institutional Review Boards of GGZ Momentum and, De Hoop GGZ and was performed in accordance with the Netherlands Code of Conduct for Research Integrity and was carried out in accordance with The Code of Ethics of the World Medical Association (Declaration of Helsinki) for experiments involving humans. All subjects provided written informed consent. Written informed consent was obtained from all participants for the publication of any potentially identifiable images or data included in this article.

## Author contributions

AK developed the study concept together with JO. AK, LG, RL, and JO contributed to the study design and performed the data analysis and interpretation, and critically revised all versions of the paper. Data collection was performed by AK, LG, and RL. All authors contributed to the article and approved the submitted version.

## Funding

AK was supported by a fellowship grant from the Netherlands Organization for Health Research and Development (ZonMw), grant number 636320001. JO was supported by the Ophelia research project, ZonMw grant number: 636340001 and Horizon 2020 project YOUTH-GEMs, grant number 101057182.

## Conflict of interest

AK was employed by GGZ Noord-Holland-Noord. LG was employed by Arkin (Netherlands). RL was employed by GGZ De Hoop.

The remaining author declares that the research was conducted in the absence of any commercial or financial relationships that could be construed as a potential conflict of interest.

## Publisher’s note

All claims expressed in this article are solely those of the authors and do not necessarily represent those of their affiliated organizations, or those of the publisher, the editors and the reviewers. Any product that may be evaluated in this article, or claim that may be made by its manufacturer, is not guaranteed or endorsed by the publisher.
