# Supplementary figures and images for: Clinician and patient perspectives on the ontology of mental disorder: a qualitative study
Source: Front Psychiatry. 2023 May 12;14:1081925. doi: 10.3389/fpsyt.2023.1081925 (PMC10213209; doi:10.3389/fpsyt.2023.1081925)

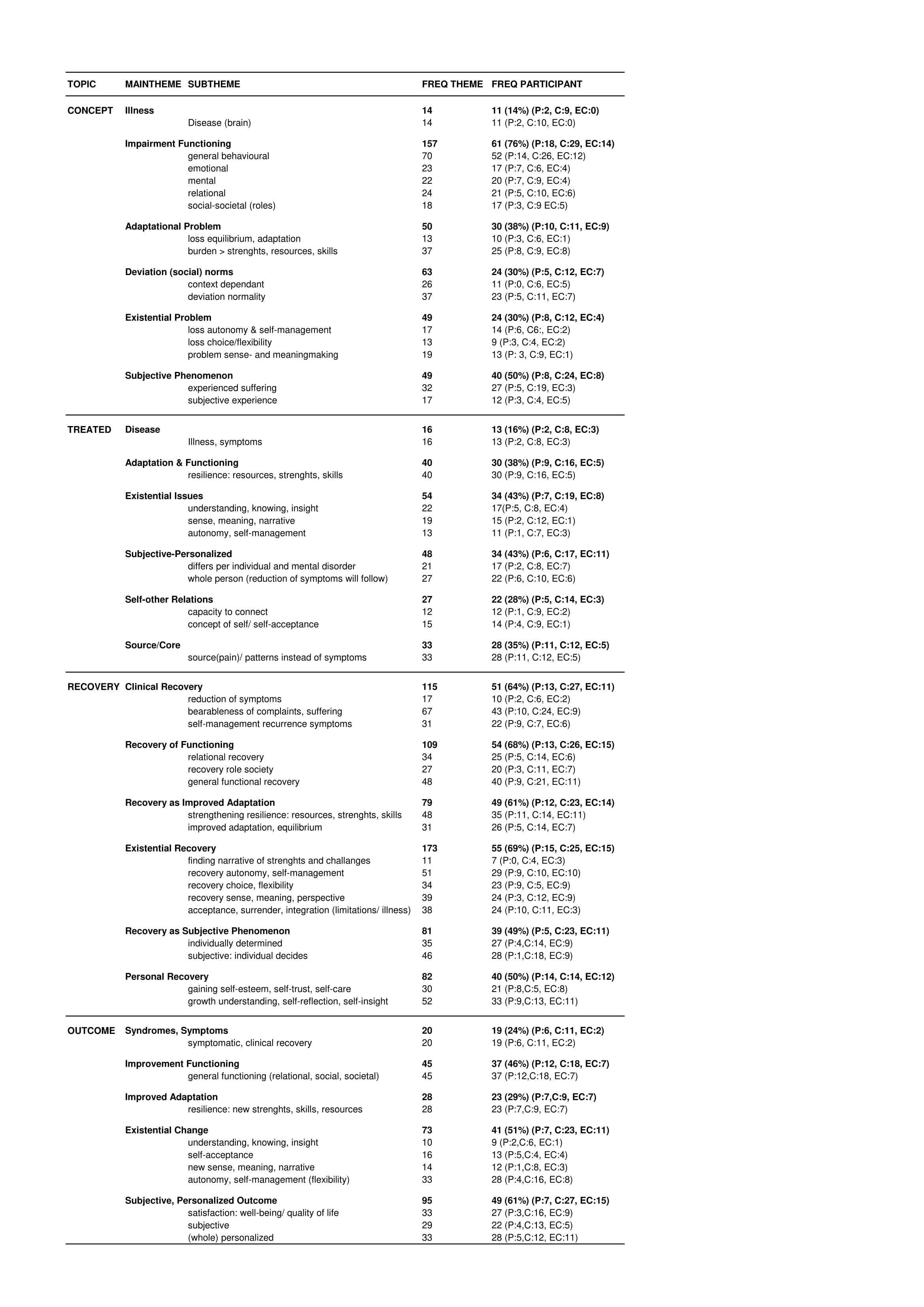

Supplement: Supplementary file 1 [file Image_1.JPEG]

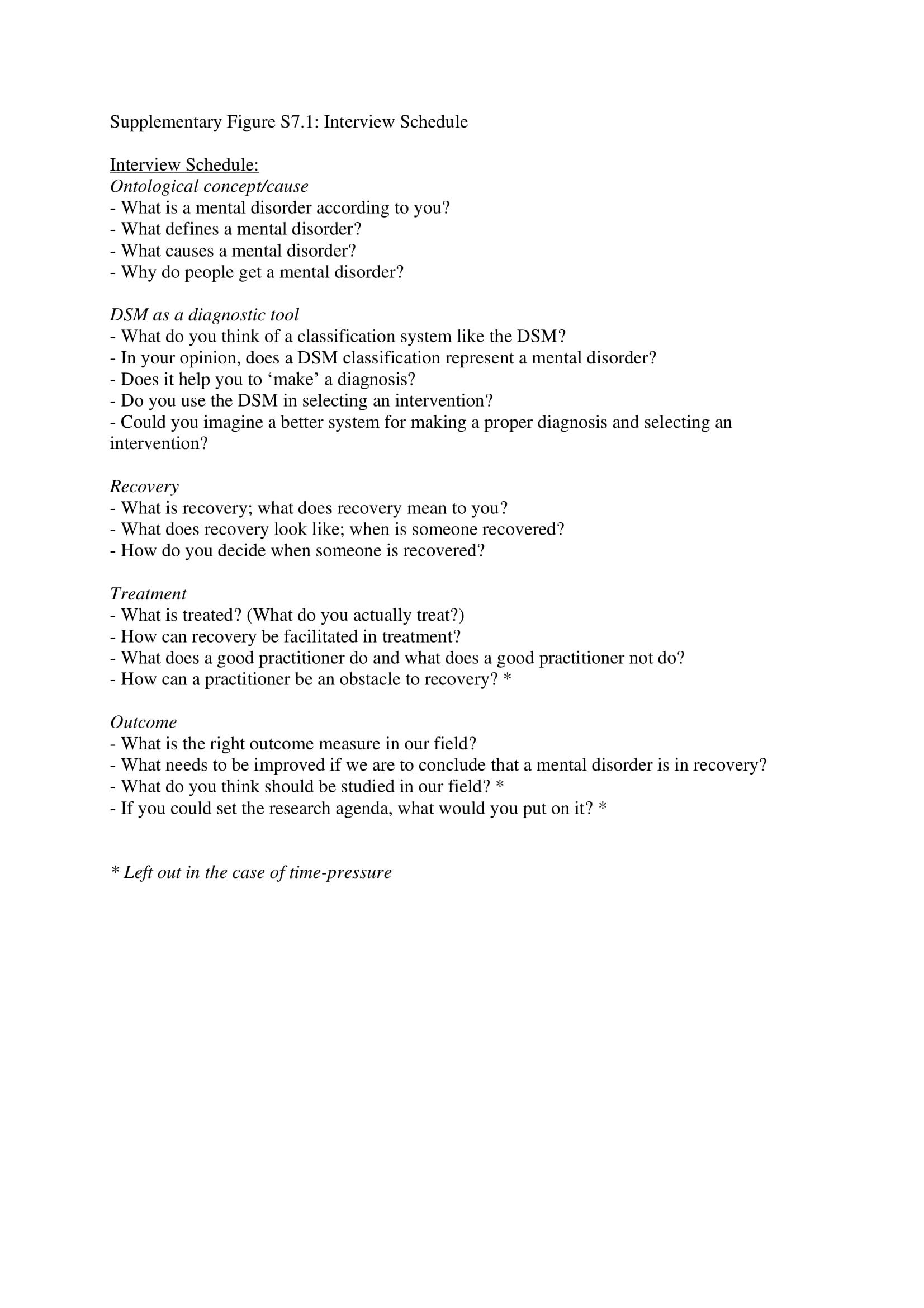

Supplement: Supplementary file 2 [file Image_2.JPEG]
